# Machine learning-driven identification of key chemical determinants of tobacco leaf sensory irritation

**DOI:** 10.3389/fchem.2026.1893276

**Published:** 2026-07-30

**Authors:** Wenting Li, Xuehui Sun, Zhe Jin, Bin Peng, Jizhao Guo, Ran Wan, Song Yang, Junwei Guo, Hongbo Wang, Meijuan Fan, Feng Li, Cong Nie, Le Zhao, Chao Wang

**Affiliations:** 1 Key Laboratory of Tobacco Chemistry, Zhengzhou Tobacco Research Institute of China National Tobacco Corporation (CNTC), Zhengzhou, China; 2 Technology Center, China Tobacco Jilin Industrial Co., Ltd., Changchun, China

**Keywords:** chemical components, feature selection, irritation, random forest, relieff, tobacco

## Abstract

**Introduction:**

To identify the key chemical components affecting the sensory irritation of tobacco leaves, a binary classification model for high and low irritation was constructed based on 78 chemical components and sensory evaluation scores of 353 tobacco leaf samples.

**Methods:**

First, the median absolute deviation (MAD) method was applied to remove outliers from the high- and low-irritation samples. Then, the ReliefF algorithm was applied for dimensionality reduction, selecting 33 core features to eliminate data redundancy. Using the selected features, a random forest (RF) algorithm was employed to build the classification model, and the optimal number of decision trees was determined to be 60.

**Results:**

The ReliefF-RF model achieved an accuracy of 84.38% on an independent test set, with precision, recall, and F1-score all at 86.49%, outperforming the original RF model as well as other machine learning models such as support vector machine (SVM) and k-nearest neighbors (KNN). Through feature importance evaluation, eight key chemical indicators were identified: total nitrogen, total alkaloids, cryptochlorogenic acid, oleic acid + linolenic acid, reducing sugar, sugar-nitrogen ratio, Fru-Asp, and neochlorogenic acid.

**Discussion:**

SHapley Additive exPlanations (SHAP) analysis revealed that higher levels of nitrogenous compounds were strongly associated with increased irritation, whereas elevated levels of sugar components, specific organic acids, and amino acid derivatives were associated with reduced irritation. Notably, Fru-Asp exhibited a complex non-linear response, where both extremely high and low levels contributed to higher irritation. This study provides a useful reference and data support for the targeted regulation of cigarette irritation.

## 1 Introduction

Tobacco, a globally cultivated non-food cash crop, is grown in over 125 countries and regions, generating hundreds of billions of dollars in annual revenue. Compared to many food crops, tobacco cultivation offers farmers significantly higher economic returns (Kakar et al., 2020). In China, to enhance cigarette quality, tobacco leaves purchased from farmers undergo a series of processes including redrying, fermentation, cutting, rolling, and packaging to produce finished cigarettes. The quality of finished cigarettes directly determines their market value and consumer acceptance (Ke et al., 2026; Yang et al., 2023). Quality inspection and evaluation of cigarettes mainly relies on sensory evaluation (Xu et al., 2026; Yan et al., 2021). This method uses the sensory systems of the evaluators, including vision, smell, touch, taste, and hearing, to quantify, analyze, and interpret product quality characteristics. Evaluation dimensions typically include luster, aroma, harmony, off-flavor, aftertaste, and irritation of cigarettes (Zhang et al., 2019). Among these, irritation refers to the negative sensory experiences such as burning, dryness, pungency, coughing, and residual aftertaste in the mouth, throat, and nasal cavity of smokers when smoking cigarettes, and is a core indicator of cigarette sensory quality (Tian et al., 2021). The perception of sensory irritation is mediated by chemosensory receptors such as TRPA1, TRPV1, and nicotinic acetylcholine receptors (nAChRs), which respond to electrophilic irritants, heat, and nicotine, respectively, thereby eliciting burning and pungent sensations (Carstens and Carstens, 2022; Kichko et al., 2018). Studies have shown that volatile carbonyl compounds, phenols, free nicotine, ammonia, and other substances in smoke are the main chemical sources of sensory irritation, and high-temperature incomplete combustion significantly exacerbates the generation of irritants (Kärkelä et al., 2022; Kichko et al., 2015; Willems et al., 2006).

Factors affecting the generation of irritants mainly include tobacco raw materials (Fu et al., 2026), cigarette design parameters (Shi et al., 2013), additives and processing technology, and combustion and smoking conditions (Zhao et al., 2021). Regarding tobacco raw materials, different types, varieties, parts, maturity, grades, and blends all affect the generation of irritants. Different tobacco types, such as flue-cured tobacco, burley tobacco and oriental tobacco have significantly different chemical compositions (Schwanz et al., 2019; Wu et al., 2026). For example, burley tobacco has a higher nitrogen content, producing more alkaline substances (such as ammonia) after combustion, leading to stronger throat irritation (Li et al., 2018). Different varieties of the same tobacco type have different contents of sugars, alkaloids, nitrogenous compounds, and minerals, thereby affecting irritant generation (Ke et al., 2026). The tobacco leaf position also plays an important role; upper leaves are usually thicker, with higher contents of nicotine, nitrogenous compounds, and minerals, and a higher degree of incomplete combustion, producing more irritants (such as ammonia, certain aldehydes, phenols), resulting in the strongest irritation; middle leaves have relatively coordinated chemical components and usually lower irritation; lower leaves are thinner, with relatively higher sugars and lower nitrogenous compounds, and although irritation is low, there may be insufficient smoke volume. In addition, immature leaves have heavy green off-flavor and more irritants (Yang et al., 2018). Low-grade leaves usually contain more stems and broken leaves, have poor combustibility, and are prone to off-flavors and irritants. Cigarette blending is a mixture of leaves of different grades, types, and origins, aiming to balance flavor and reduce irritation. If the proportion of highly irritating leaves (such as some upper leaves, burley tobacco) is too high, the overall irritation will be significantly increased. Cigarette sensory irritation (such as throat burning sensation, oral roughness, nasal stinging sensation) is closely related to the chemical composition of tobacco raw materials and the smoke components generated from their combustion. Numerous studies have shown that nitrogenous compounds are significantly positively correlated with irritation (Wang M.F. et al., 2010). The higher the total nitrogen and nicotine content in tobacco, the stronger the irritation (Li Y. H. et al., 2024). Nicotine directly triggers burning pain in the mouth and throat by activating trigeminal nociceptors (Carstens and Carstens, 2022). Upper leaves generally have stronger irritation than middle and lower leaves due to their higher total nitrogen and nicotine content. The sugar-to-nitrogen ratio and sugar-to-nicotine ratio are significantly negatively correlated with irritation. A high sugar-to-nitrogen ratio helps neutralize alkaline irritants (such as ammonia) produced by nitrogenous compounds, thereby reducing throat scratchiness. Total sugar and reducing sugar generate acidic components during combustion, which can neutralize alkaline irritants; therefore, total sugar content is negatively correlated with irritation and significantly improves off-flavor. Among mineral elements, potassium and chlorine have a more prominent impact. High potassium content promotes complete combustion, reducing irritating substances such as tar and aldehydes (Xu et al., 2024; Yamamoto et al., 2015); high chlorine content tends to cause incomplete combustion, increasing aldehyde and phenol generation (Tiecher et al., 2023). Both are negatively correlated with irritation, but potassium contributes more. An appropriate potassium-to-chlorine ratio optimizes combustibility and reduces carbon monoxide and aldehyde release. Organic acids also participate in the regulation of irritation. Volatile acids (e.g., valeric acid) have a high transfer rate when added via breakable capsules and can mask irritation to some extent, but excessive amounts may cause acid irritation; non-volatile acids (e.g., palmitic acid, stearic acid) are negatively correlated with irritation in cigar filler, but high-temperature pyrolysis may generate small-molecule irritating acids (Geng et al., 2023). However, existing studies are mostly limited to elucidating simple correlations between individual chemical components and cigarette irritation, and have not yet systematically established quantitative association models linking irritation to chemical composition.

In recent years, a substantial body of research has applied interpretable machine learning to the modeling and analysis of tobacco sensory quality. For instance, Zang et al. (2026) employed machine learning algorithms to construct quantitative prediction models for nutty, caramel-sweet, and mellow-sweet flavor notes based on metabolomics data from 21 tobacco varieties, achieving R^2^ values of 0.86–0.90. Through SHapley Additive exPlanations (SHAP) analysis, they further revealed the contribution of metabolites such as sugars and organic acids to the aroma characteristics of flue-cured tobacco. Bie et al. (2022) measured multiple chemical components in Shandong tobacco leaves and combined extreme gradient boosting (XGBoost) to build a predictive model for tobacco quality grades, which reached an accuracy of 85%. Their SHAP analysis identified seven chemical indicators, including the acid-to-phenol ratio and sucrose, as major contributors to tobacco quality. Collectively, these studies demonstrate that interpretable machine learning can quantitatively evaluate the influence of chemical components on tobacco sensory quality via feature contribution analysis, thereby providing a prioritized ranking of key chemical indicators for raw material selection and formula design.

Although there have been many studies on the relationship between chemical components and irritation, most existing research remains at the level of qualitative description or simple correlation analysis, lacking systematic quantitative associations and predictive models. Based on chemical composition data and sensory irritation scores, this study constructs a high/ low irritation classification model and, combined with interpretable methods such as feature importance analysis, identifies key chemical components affecting sensory irritation. This study aims to develop a transparent predictive and interpretative framework linking chemical components to sensory irritation, thereby providing a data-driven theoretical basis and specific chemical indicators for cigarette raw material selection, formula optimization, and irritation regulation.

## 2 Materials and methods

### 2.1 Materials and instruments

Materials: A total of 353 tobacco leaf raw materials collected from different production areas in China and other countries were used. The origin information of the raw materials is shown in Table 1.

**TABLE 1:** Origin information of samples.

Country	Province	Number of samples
Argentina	—	3
Brazil	—	15
Zimbabwe	—	14
Malawi	—	3
American	—	12
China	Fujian	41
	Guizhou	23
	Henan	46
	Heilongjiang	6
	Hunan	46
	Jilin	15
	Liaoning	2
	Neimenggu	1
	Shandong	2
	Shanxi	3
	Yunnan	121

Instruments: Antaris II Fourier Transform Near-Infrared Spectrometer (equipped with an integrating sphere diffuse reflectance sampling system, Thermo Scientific, United States); LC-2232 Adjustable Temperature Oven (Espec Environmental Equipment Co., Ltd., Shanghai, China); ZM200 grinder (Retsch, Germany); BSA124S Analytical Balance (readability: 0.1 mg, Sartorius, Germany); 1200 Series Liquid Chromatograph (Agilent Technologies, United States); AB Sciex TripleTOF™ 4600 Mass Spectrometry System (Applied Biosystems, United States).

### 2.2 Methods

#### 2.2.1 Sample pretreatment

All tobacco samples were dried according to the China’s tobacco industry standard YC/T 31–1996, then ground using a grinder equipped with a 60-mesh (0.250 mm aperture) sieve, homogenized, and stored in sealed bags. The moisture content of the samples was determined to be 6%–8% using the same China’s industry standard YC/T 31–1996.

#### 2.2.2 Near-infrared spectral acquisition

Spectral acquisition parameters: Spectra were collected using a near-infrared spectrometer operating in the 4000–10000 cm^-1^ wavenumber range, with 64 scans per measurement, at a spectral resolution of 8 cm^-1^ and two spectral replicates per sample. A correlation coefficient threshold of >0.9999 was applied to ensure consistency between replicate spectra. Environmental conditions were maintained at 20 °C–27 °C and 30%–50% relative humidity. Background spectra were collected every 20 min.

Spectral preprocessing method (Li et al., 2025): Prior to modeling, multiplicative scatter correction (MSC) was applied to mitigate scattering effects caused by uneven particle size and distribution in the samples (Helland et al., 1995). To minimize the noise amplification inherent in derivative calculations, Savitzky-Golay convolution smoothing (window size: 17 points; polynomial order: 2) was performed prior to the first-derivative transformation. This step also eliminated constant baseline offsets in the spectra (Savitzky and Golay, 1964).

#### 2.2.3 Determination of chemical component contents

The near-infrared analysis platform system of the China National Tobacco Corporation was used to predict 70 chemical indicators, including routine chemical components, cations and anions, polyphenols, polyacids and higher fatty acids, amino acids, and Amadori compounds, basically covering the macro and semi-micro chemical components that affect tobacco leaf quality and style (Li et al., 2025). Calibration model performance (R^2^, RMSEP and RPD) for these 70 indicators is reported in Liang et al. (2022).

In addition, the sugar-nitrogen ratio, potassium-chlorine ratio, and sugar-total amino acids (sugar-AA ratio) were obtained by ratio derivation, and the total amounts of flame-retardant anions, total polyphenols, total non-volatile acids, total amino acids (AA), and total Amadori compounds were obtained by summation. The types of chemical components and corresponding chemical components in tobacco leaves are shown in Table 2.

**TABLE 2:** Classification of chemical components in tobacco leaves.

No.	Type	Specific feature	Number of chemical components
1	Routine chemical components	Total alkaloids, reducing sugar, total sugar, total nitrogen, potassium, chlorine, starch	7
2	Cations and anions	Potassium, chlorine, sulfate, phosphate, magnesium, calcium	6
3	Polyphenols	Neochlorogenic acid, chlorogenic acid, cryptochlorogenic acid, scopoletin, rutin	5
4	Polyacids and higher fatty acids	Oxalic acid, malonic acid, succinic acid, malic acid, citric acid, vanillic acid, myristic acid, palmitic acid, linoleic acid, oleic acid + linolenic acid, stearic acid, arachidic acid	12
5	Amino acids	Aspartic acid (asp), threonine (Thr), serine (ser), asparagine (asn), glutamic acid (Glu), Glutamine (Gln), Glycine (Gly), Alanine (Ala), Valine (Val), Cystine (Cys), Methionine (Met), Isoleucine (Ile), Leucine (Leu), Tyrosine (Tyr), Phenylalanine (Phe), 4-aminobutyric acid (GABA), Lysine (Lys), Histidine (His), Tryptophan (Trp), Arginine (Arg), Proline (Pro)	21
6	Amadori compounds	N-(1-deoxy-d-glucose-1-yl) ammonia (glu-an), N-(1-deoxy-D-fructos-1-yl) aminobutyric (fru-amb), N-(1-deoxy-D-fructos-1-yl) histidine (fru-his), N-(1-deoxy-D-fructos-1-yl) proline (fru-pro), N-(1-deoxy-D-fructos-1-yl) valine (fru-val), N-(1-deoxy-D-fructos-1-yl) threonine (fru-thr), N-(1-deoxy-D-fructos-1-yl) glycine (fru-gly), N-(1-deoxy-D-fructos-1-yl) alanine (Fru-Ala), N-(1-deoxy-D-fructos-1-yl) asparagine (fru-asn), N-(1-deoxy-D-fructos-1-yl) asparticacid (fru-asp), N-(1-deoxy-D-fructos-1-yl) glutarnine (fru-gln), N-(1-deoxy-D-fructos-1-yl) glutamicacid (fru-glu), N-(1-deoxy-D-fructos-1-yl) isoleucine (fru-ile), N-(1-deoxy-D-fructos-1-yl) leucine (fru-leu), N-(1-deoxy-D-fructos-1-yl) tyrosine (fru-tyr), N-(1-deoxy-D-fructos-1-yl) phenylalanine (fru-phe), N-(1-deoxy-D-fructos-1-yl) tryptophan (fru-trp)	17
7	Others	pH, dichloromethane extract, lycopene, neophytadiene	4
8	Derivatization indexes	Sugar-nitrogen ratio, potassium-chlorine ratio, flame-retardant anions, total polyphenols, total non-volatile acids, total amino acids (AA), Sugar-AA ratio, total amadori compounds	8
Total (deduplicated)			78

#### 2.2.4 Sensory quality evaluation method

The collected tobacco leaves were rolled into cigarettes using unified auxiliary materials. The irritation of the cigarettes was evaluated according to the requirements of YC/T138–1998, using a 0–10-point scale, to obtain the sensory irritation scores of the tobacco leaf samples.

#### 2.2.5 Construction of irritation classification model and feature importance evaluation

**FIGURE 1:**
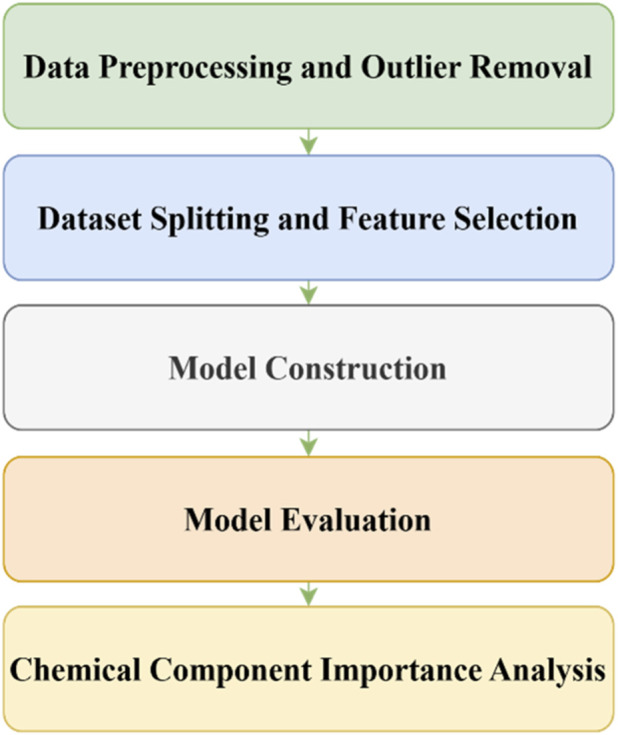
Model construction workflow.

To investigate the relationship between tobacco chemical components and irritation, a binary random forest (RF) model for high-irritation and low-irritation classes was constructed based on sensory irritation scores, and feature importance evaluation was used to identify key chemical components significantly contributing to irritation classification. This specifically includes the following five key steps (Figure 1).

##### 2.2.5.1 Data preprocessing and outlier removal

To ensure data quality and define classification targets, sensory irritation scores were categorized based on both the empirical distribution and expert panel consensus. Samples with scores ≥ 6.0 were classified as low-irritation, those with scores ≤ 5.5 as high-irritation, and samples falling within the ambiguous interval (5.5, 6.0) were excluded to enhance class separability.

To eliminate the interference of abnormal samples on subsequent modeling, outliers were removed separately for the two classes. The Euclidean distance from each sample to its class mean was calculated, and the median absolute deviation (MAD) method was used to identify and remove outliers (Leys et al., 2013). For a dataset $X_{m\times n}$ with *m* samples and *n* features, the median $median\left(X\right)$ of the data was first calculated, then the absolute deviation $\left|x_{i}-median\left(X\right)\right|$ of each data point from the median was calculated. The median of the absolute deviations is the MAD, calculated using Equation 1:

$$MAD=median\left(\left|x_{i}-median\left(X\right)\right|\right)$$ (1)

Points exceeding the median ± 3 MAD were considered outliers and removed.

##### 2.2.5.2 Dataset splitting and feature selection

The dataset was randomly split into a training set and a test set at a ratio of 7:3. Five-fold cross-validation (5-Fold) was applied on the training set to optimize model hyperparameters. Based on the optimal parameters, the training set was used to build a binary classification model for high-irritation and low-irritation tobacco leaf samples, and the test set was used to evaluate the reliability of the model.

For the small-sample, multi-feature dataset, the ReliefF feature selection algorithm was used for dimensionality reduction and screening (Robnik-Šikonja and Kononenko, 2003). The core idea of the ReliefF algorithm is: if a feature has good classification ability, samples of the same class should be close on that feature, while samples of different classes should be far apart on that feature. The specific procedure of the ReliefF algorithm is as follows: randomly select a sample *R* from the training set, find its *k* nearest neighbors $H_{j}$ (*j* = 1,2, … ,*k*) from the same class and *k* nearest neighbors $M_{j}$ from different classes. For each feature *x*, calculate the average distance $diff\left(x,R,H\right)$ between *R* and all $H_{j}$ and $M_{j}$ on *x* using Equation 2. If the average distance *avgHit* between *R* and $H_{j}$ on *x* (see Equation 3) is smaller than the average distance *avgMiss* between *R* and $M_{j}$ (see Equation 4), it indicates that the feature helps distinguish between the same class and different classes, and the weight $W\left(x\right)$ of the feature is increased; otherwise, the weight is decreased. Iterate through the training set samples and update the weights according to Equation 5:

$$diff\left(x,R,H\right)=\frac{\left|R_{x}-S_{x}\right|}{\max\left(x\right)-\min\left(x\right)}$$ (2)

$$avgHit=\frac{1}{k}\sum_{j=1}^{k}diff\left(x,R,H_{j}\right)$$ (3)

$$avgMiss=\frac{1}{k}\sum_{j=1}^{k}diff\left(x,R,M_{j}\right)$$ (4)

$$W\left(x\right)=W\left(x\right)-\frac{avgHit}{m}+\frac{avgMiss}{m}$$ (5)

where $\max\left(x\right)$ and $\min\left(x\right)$ are the maximum and minimum values of feature *x* in the training set, and *m* is the total number of samples in the training set. Finally, the classification ability of each feature is evaluated according to its average weight, and features with the highest weights are selected to form the core feature subset.

##### 2.2.5.3 Model construction

The RF algorithm (Breiman, 2001) was used to build the binary classification model. The RF model randomly draws with replacement draws training set samples and their corresponding features to form multiple different training subsets. Multiple independent decision trees are built for each subset, and the number of decision trees is determined iteratively by the out-of-bag (OOB) error. The OOB error is an indicator for evaluating model performance during classification model training, calculated using Equation 6 as follows:

$$out-of-bagerror=\frac{1}{N}\sum_{i=1}^{N}I\left(\hat{y_{i}}\neq y_{i}\right)$$ (6)

Each decision tree independently classifies the tobacco leaf data as high or low irritation, and the final classification prediction is determined by majority voting of the prediction results from all decision trees.

##### 2.2.5.4 Model evaluation

The trained RF model was applied to the test set samples partitioned in Step 2 to classify tobacco leaves into high- or low-irritancy categories. A confusion matrix was constructed based on the true classes of the samples and the classes predicted by the model. The confusion matrix of the classification results is presented in Table 3.

**TABLE 3:** Binary classification confusion matrix.

True class	Predicted	
	Low irritation	High irritation
Low irritation	TP (Number of samples correctly predicted as low irritancy)	FN (Number of samples incorrectly predicted as high irritancy)
High irritation	FP (Number of samples incorrectly predicted as low irritancy)	TN (Number of samples correctly predicted as high irritancy)

Based on the confusion matrix, accuracy, precision, recall, and F1-score were calculated to evaluate the classification model performance. The closer these indicators are to 1, the better the model classification effect. The calculation formulas for each evaluation indicator are shown in Equations 7-10:

$$Accuracy=\frac{TP+TN}{TP+TN+FP+FN}$$ (7)

$$Precision=\frac{TP}{TP+FP}$$ (8)

$$Recall=\frac{TP}{TP+FN}$$ (9)

$$F1-Score=2\times \frac{Precision\times Recall}{Precision+Recall}$$ (10)

##### 2.2.5.5 Chemical component importance analysis

RF is an ensemble of multiple decision trees. During the construction of the classification model, feature importance evaluation is achieved by quantifying the purity increase (or error decrease) brought by that feature in node splits across all decision trees. By arithmetically averaging and normalizing the contribution of each feature across all decision trees, a comprehensive importance ranking of all chemical indicators is generated, thereby extracting the chemical components that play a key role in determining high/low irritation of samples (Breiman, 2001).

### 2.3 Data processing methods

All data analysis procedures were conducted using MATLAB R2022b (MathWorks, United States).

## 3 Results and discussion

### 3.1 Sample screening and data distribution

**FIGURE 2:**
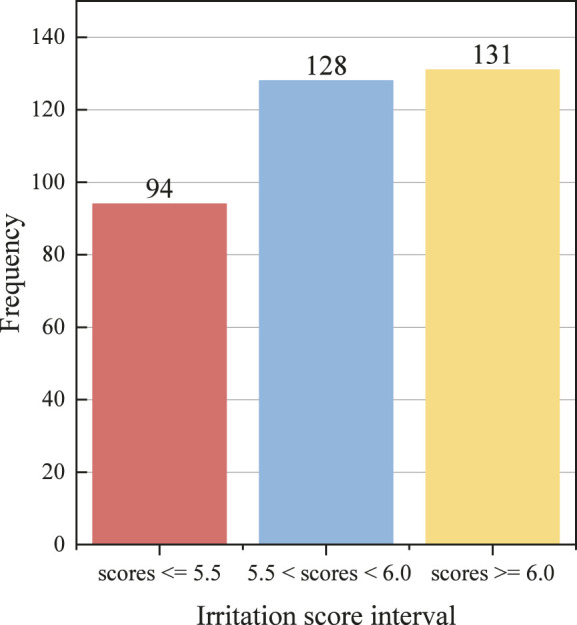
Distribution of sample sizes across irritation score intervals.

The evaluation panel consisted of trained assessors with extensive experience in tobacco sensory evaluation. To examine the consistency among the expert sensory panel, Kendall’s coefficient of concordance was applied to the irritation scores of 353 samples, yielding a coefficient of 0.82 (P = 0.0032), indicating that the scoring results were significantly consistent. Figure 2 presents the distribution of sensory irritation scores, showing 94 high-irritation, 128 low-irritation, and 131 fuzzy-interval samples. Outliers in the chemical components of the high- and low-irritation classes were identified and removed using the MAD method on Z-score standardized data.

To determine the optimal threshold factor λ for the MAD method, we evaluated the classification performance of the ReliefF-RF model under four discrete λ values (1.0, 2.0, 3.0, and 4.0). Table 4 showed that λ = 1.0 gave the highest accuracy (93.18%) but retained only 147 samples, causing excessive information loss. As λ increased from 2.0 to 4.0, both accuracy and F1-score remained stable within 83%–85% and 0.85–0.87, respectively, while the number of retained samples gradually rose. At λ = 3.0, the model retained 214 samples with an accuracy of 84.38% and an F1-score of 0.8649, and this value aligns with the commonly accepted statistical criterion for MAD-based outlier detection (Leys et al., 2013). Therefore, λ = 3.0 was adopted as the threshold factor.

**TABLE 4:** Model performance under different MAD threshold factors (λ).

Threshold factor (λ)	Samples retained	Accuracy (%)	F1-score
1	147	93.18	0.9434
2	197	84.75	0.8696
3	214	84.38	0.8649
4	218	83.08	0.8493

**FIGURE 3:**
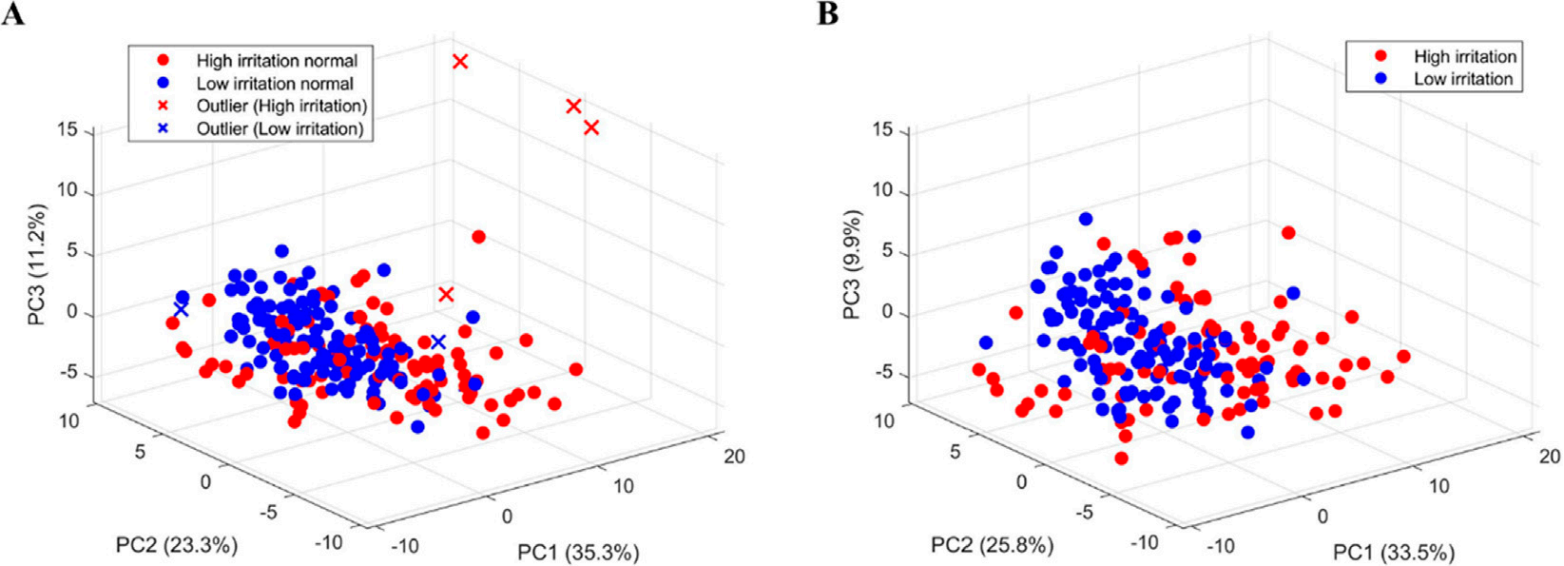
PCA score plots before and after outlier removal **(A)** Before outlier removal; **(B)** After outlier removal.

Subsequently, PCA was performed, and the score plots before and after outlier removal are displayed in Figure 3. Four outliers were detected in each class, leaving 90 high- and 124 low-irritation samples after removal. Moreover, Figure 3 reveals a substantial overlap in the projection distributions of the two classes, indicating that these two types of tobacco leaves are not effectively distinguishable in the low-dimensional linear space after PCA dimensionality reduction.

### 3.2 Chemical feature screening based on ReliefF algorithm

**FIGURE 4:**
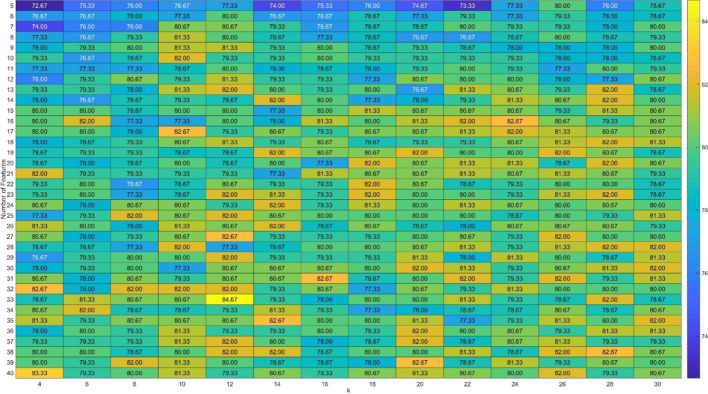
Heatmap of 5-fold cross-validation accuracy under different parameter combinations.

The ReliefF algorithm was used to screen the initial 78 chemical features. The number of neighbors (*k*) and the number of retained features (*num_features*) are two key hyperparameters affecting the screening effect. To ensure the reliability and stability of feature selection, 5-fold cross-validation was performed within the training set, simultaneously optimizing *k* (range 4–30, step 2) and *num_features* (range 5–40, step 1), and the accuracy of the RF classification model on the validation set was used as the evaluation metric. The heatmap of the 5-fold cross-validation average accuracy under different parameter combinations is shown in Figure 4. Warmer colors (more yellow) in the figure represent higher model accuracy. The results showed that the model performance was optimal when *k* = 12 and *num_features* = 33, with the 5-fold cross-validation average accuracy reaching 84.67%. The 33 features screened by weight magnitude included total alkaloids, lycopene, oleic acid + linolenic acid, total nitrogen, vanillic acid, Fru-Asp, myristic acid, sulfate, potassium, scopoletin, Fru-Val, linoleic acid, flame-retardant anions, Fru-Amb, neophytadiene, rutin, phosphate, reducing sugars, oxalic acid, Fru-Ile, pH, sugar-nitrogen ratio, malonic acid, dichloromethane extract, total sugar, cryptochlorogenic, Gly, Fru-Leu, magnesium, Fru-Pro, Fru-Phe, Calcium, and Fru-Thr.

### 3.3 Hyperparameter optimization of the RF model

**FIGURE 5:**
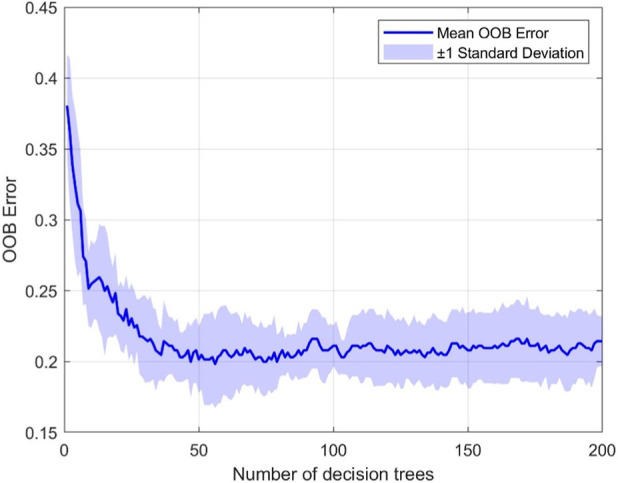
RF OOB error convergence curve under five-fold cross-validation.

When constructing the RF classification model, increasing the number of decision trees can improve model generalization and prediction accuracy, but too many trees significantly increase computational cost. The relationship between the number of decision trees and the OOB error in the high/low irritation binary classification model is shown in Figure 5. As the number of decision trees increases, the OOB error decreases rapidly, and when the number of decision trees ≥ 50, the OOB error essentially no longer changes. Considering that as the number of decision trees increases, the model construction time and computational cost increase accordingly, and the interpretability of the model decreases, while also considering error stability, the number of decision trees was set to 60.

### 3.4 Performance evaluation of the irritation classification model

**FIGURE 6:**
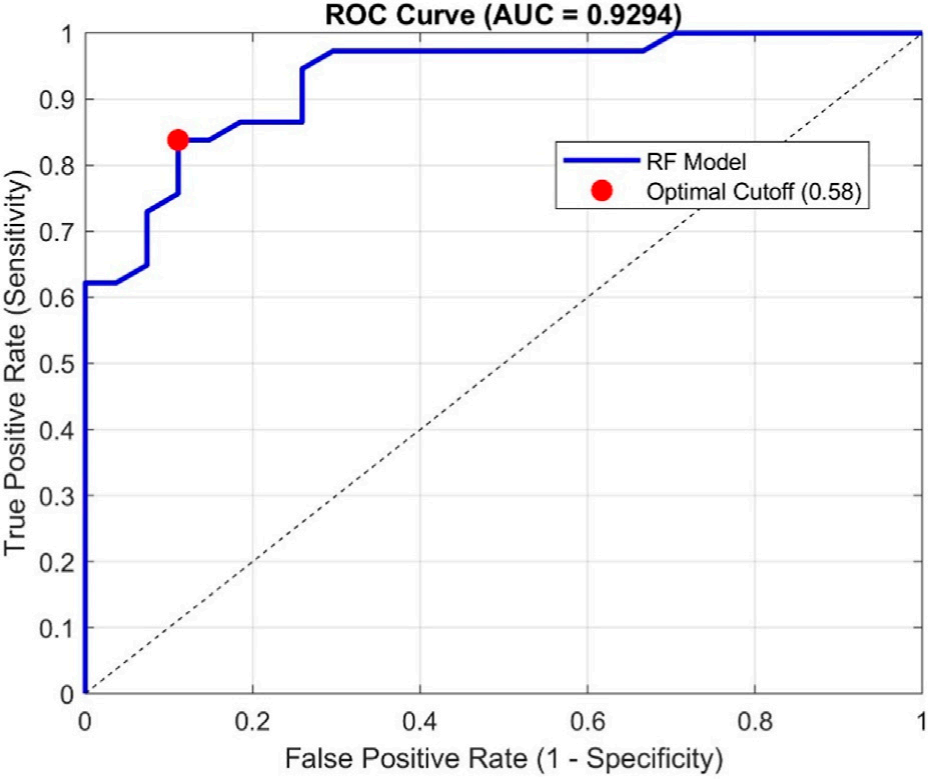
ROC curve of the ReliefF-RF model for distinguishing sensory irritation levels.

The performance of the ReliefF-RF model was rigorously evaluated via 5-fold cross-validation and an independent test set. In cross-validation, the model achieved a mean accuracy of 84.67%. When applied to the hold-out test set, the model yielded an accuracy of 84.38%, with corresponding precision, recall, and F1-score of 86.49%, indicating balanced predictive power for both classes (Table 5). Receiver operating characteristic (ROC) analysis further demonstrated robust discriminative ability, with an outstanding AUC of 0.9294 (Figure 6). The optimal probability threshold was determined to be 0.58 by maximizing the Youden index, facilitating practical classification of sensory irritation levels.

**TABLE 5:** Classification confusion matrix of the ReliefF-RF model on the independent test set.

True class	Predicted	
	Low irritation	High irritation
Low irritation	22	5
High irritation	5	32

**FIGURE 7:**
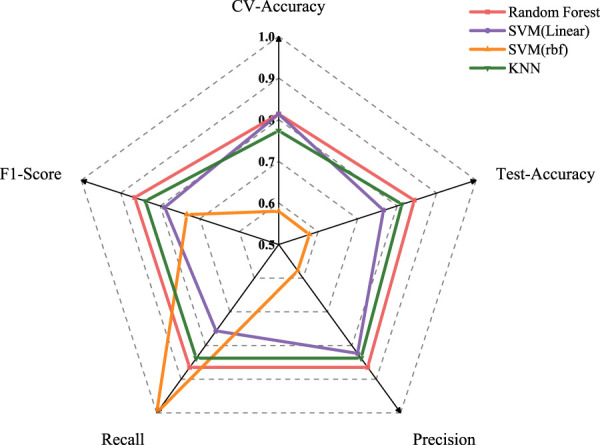
Radar chart comparing performance evaluation metrics of different machine learning classification models.

To assess the efficacy of feature engineering, we compared the ReliefF-RF with the baseline RF model without feature pre-screening. The latter exhibited a lower cross-validation accuracy of 79.33%, confirming that ReliefF-based selection effectively eliminates redundant features and enhances both classification performance and generalization. Furthermore, we extended the comparison to other conventional machine learning classifiers, including SVM (with linear and RBF kernels) and KNN, with their respective metrics summarized in the radar chart (Figure 7). To provide a more rigorous benchmark, we also incorporated advanced gradient boosting algorithms, namely XGBoost and LightGBM. Interestingly, both boosting methods achieved identical classification outcomes on the test set—matching the accuracy (84.38%) and confusion matrix of the ReliefF-RF model. This parity suggests that while the proposed model attains performance on par with state-of-the-art tree-based ensembles, it retains the inherent advantages of RF in interpretability and computational efficiency. Overall, the comprehensive comparative results underscore the superiority and robustness of the ReliefF-RF model across multiple performance dimensions.

### 3.5 Importance analysis of key chemical components

**FIGURE 8:**
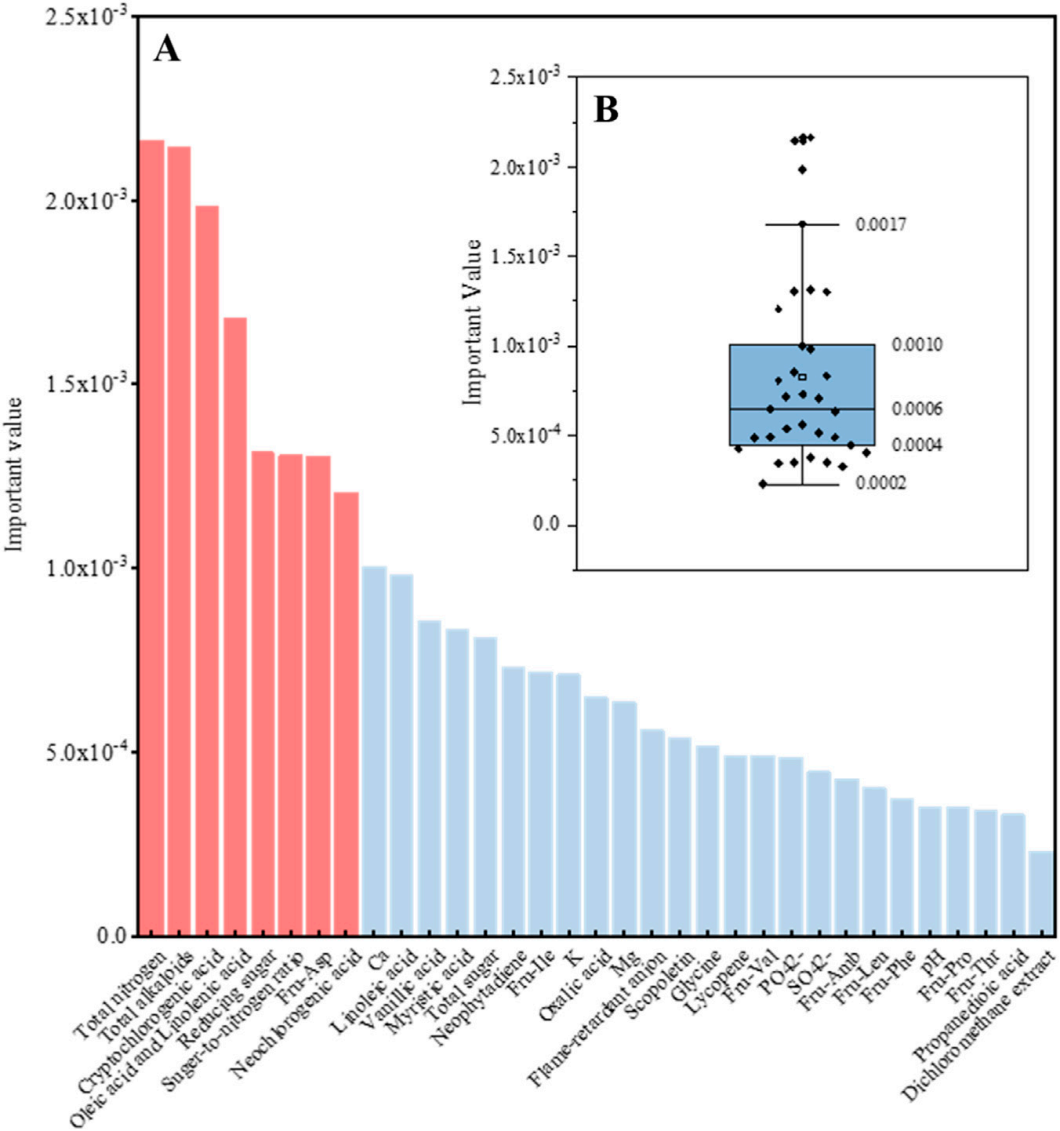
Feature importance analysis of chemical components based on the ReliefF algorithm **(A)** distribution of feature importance values; **(B)** boxplot of importance threshold division).

The statistical distribution of importance values for the 33 chemical features screened by the ReliefF algorithm showed that the upper quartile of the importance value distribution was 0.0010. Thus, all chemical indicators were divided into key indicators and general indicators based on importance values. The key indicators included a total of eight components: total nitrogen, total alkaloids, cryptochlorogenic acid, oleic acid + linolenic acid, reducing sugars, sugar-to-nitrogen ratio, Fru-Asp, and neochlorogenic acid; the general indicators included 25 chemical components such as Ca, linoleic acid, and vanillic acid (Figure 8).

**FIGURE 9:**
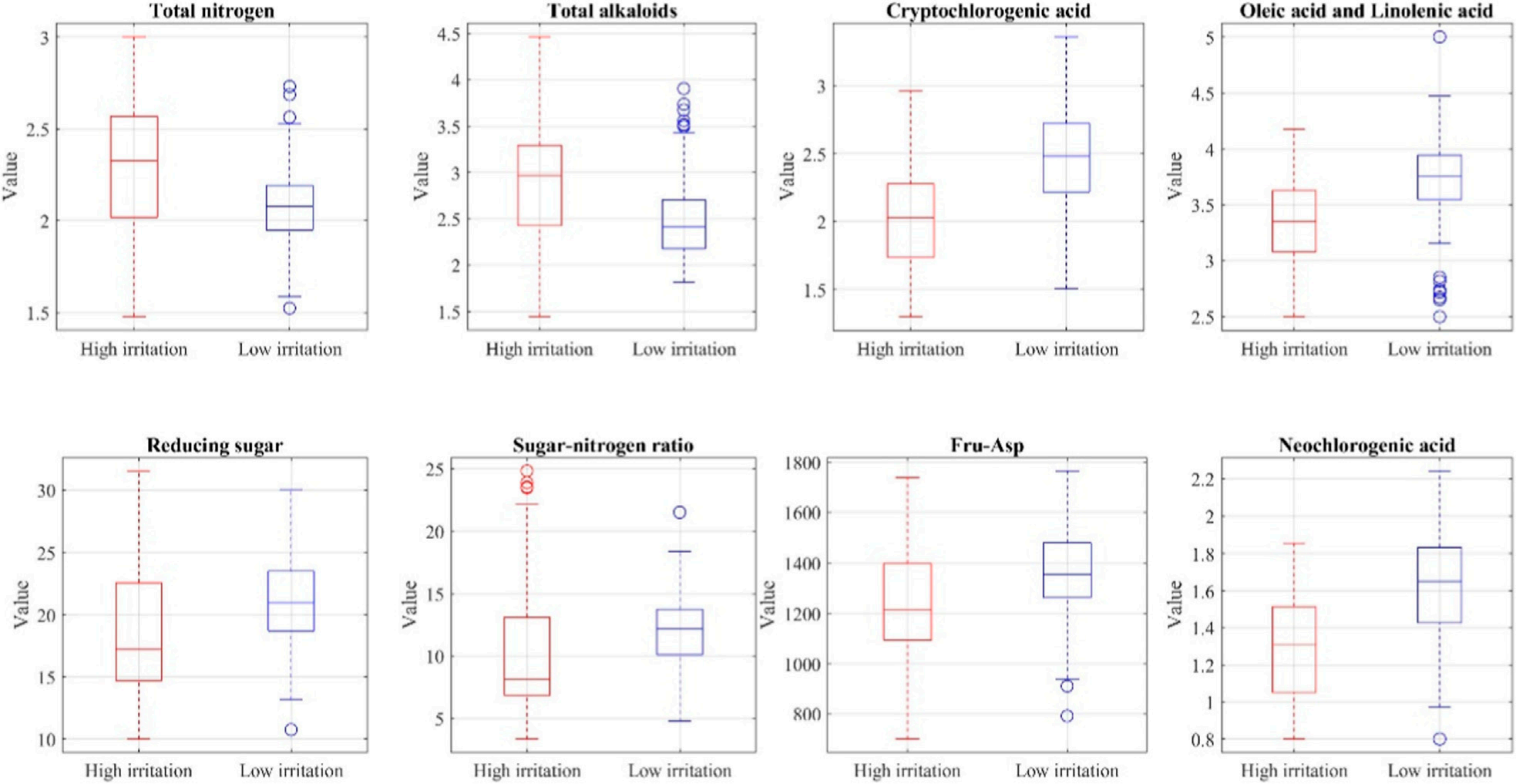
Boxplots of the distribution of eight key chemical component features in high- and low-irritation samples.

Figure 9 shows the distribution of these eight key indicators in high- and low-irritation tobacco leaf samples. The results showed that the contents of total nitrogen and total alkaloids are significantly higher in high-irritation samples than in low-irritation samples; whereas the contents of cryptochlorogenic acid, oleic acid + linolenic acid, reducing sugar, sugar-nitrogen ratio, Fru-Asp, and neochlorogenic acid are relatively higher in low-irritation samples.

**FIGURE 10:**
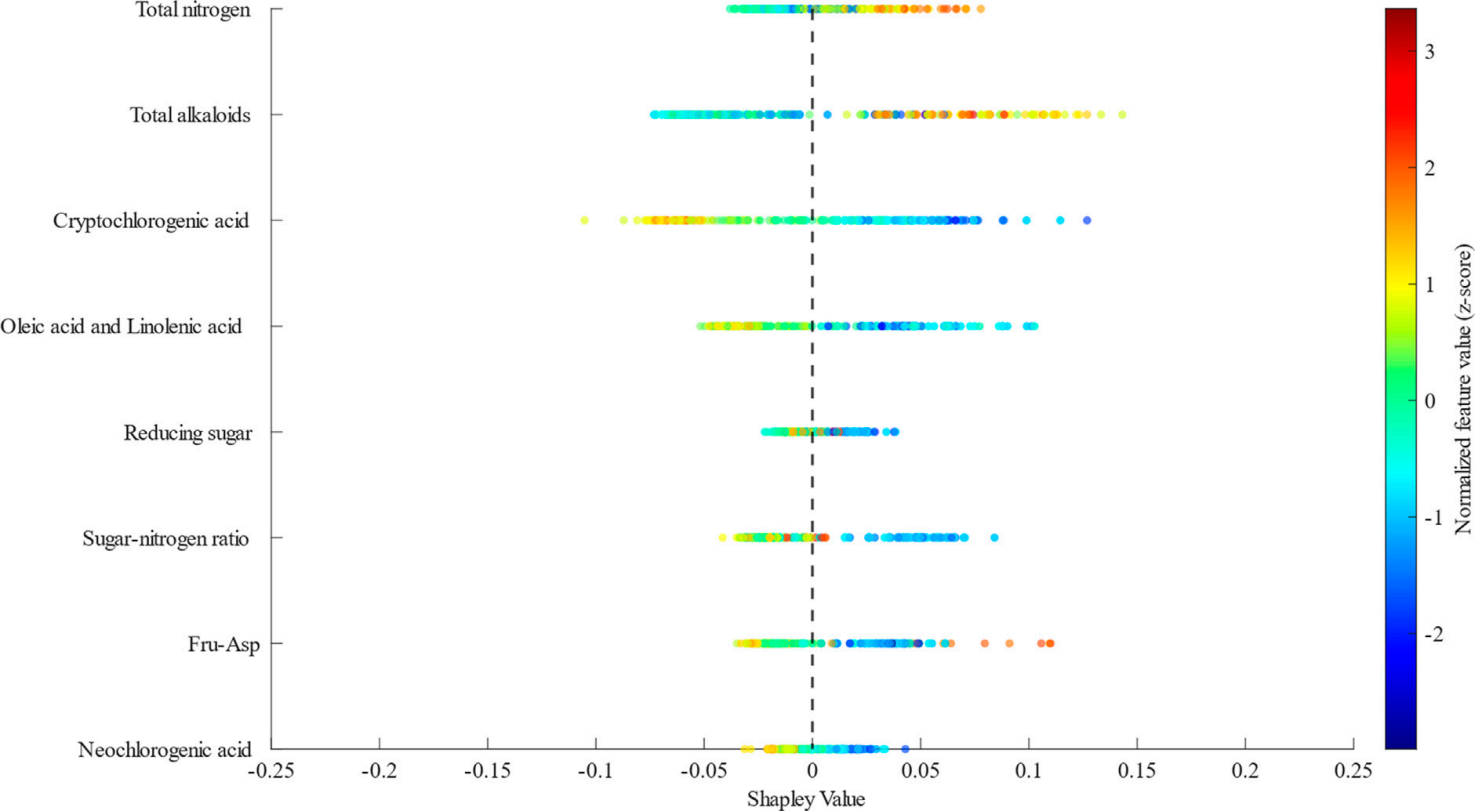
SHAP values of eight key indicators for predicting high irritation.

To further elucidate the formation mechanism of high irritation in tobacco leaves, SHAP analysis was performed on the above eight indicators, and the results are presented in Figure 10. As shown, increases in total alkaloids and total nitrogen lead to higher Shapley values, thereby significantly driving the model to classify tobacco leaves as high-irritation. In contrast, increases in cryptochlorogenic acid, oleic acid + linolenic acid, reducing sugar, sugar-nitrogen ratio, and neochlorogenic acid result in lower Shapley values, tending to reduce the probability of being classified as high-irritation. Notably, the effect of Fru-Asp on model predictions exhibited a non-linear U-shaped trend: both low and high levels of Fru-Asp drive the model toward a “high-irritation” classification. These findings suggest that an increase in nitrogen-containing compounds (e.g., total alkaloids, total nitrogen) may directly enhance tobacco leaf irritation; in contrast, an increase in sugars (e.g., reducing sugars), certain organic acids, and amino acid derivatives helps to neutralize or reduce irritation. Previous studies have shown that, compared with low-quality flue-cured tobacco, high-quality flue-cured tobacco leaves typically have higher total sugar and lower total nitrogen (Liu et al., 2025). Rogers et al. pointed out that lipid compounds such as higher fatty acids in tobacco leaves can mellow smoke, with linolenic acid being a representative component (Hu et al., 2023; Rogers and Mitchem, 1976). Furthermore, Fru-Asp, an Amadori compound present in relatively high amounts in tobacco leaves (Li et al., 2025), is an important type of aroma precursor. During cigarette smoking, it can generate numerous aroma products through the Maillard reaction, thereby modifying and mellowing the smoke and reducing irritation (Li et al., 2025; Ren et al., 2024). However, Li X. Y. et al. (2024) found that the relationship between most Amadori compounds and sensory quality is not strictly linear and positive; instead, excessive contents may conversely be detrimental to tobacco leaf quality.

**FIGURE 11:**
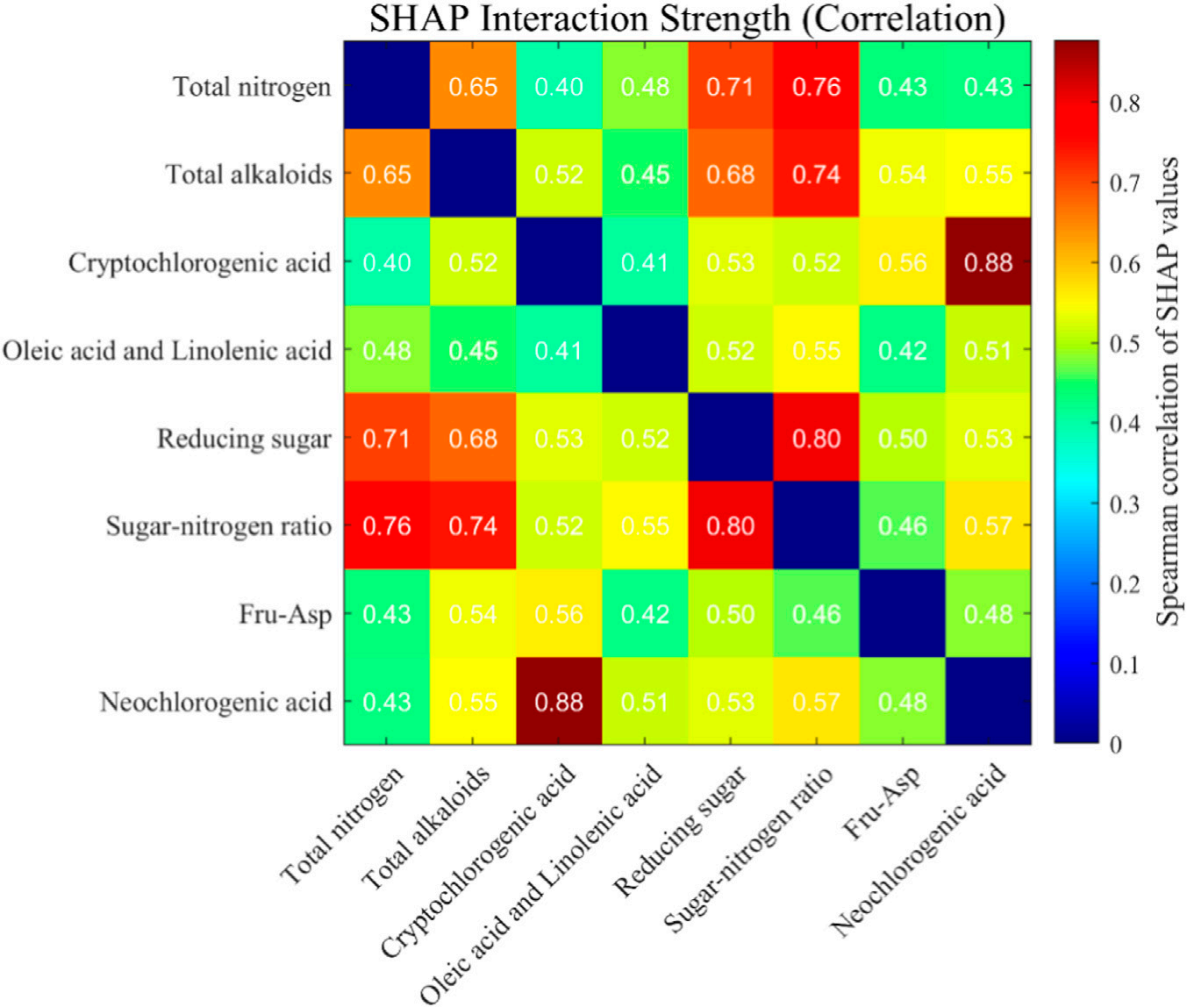
Spearman correlation heatmap of SHAP values for the eight key chemical components.

To quantify the interactions among key features, Spearman correlation coefficients of SHAP values for the eight core features were calculated, with the results presented in Figure 11. As shown, the correlation coefficients between saccharide-related indicators (reducing sugars, sugar-to-nitrogen ratio) and nitrogen-related indicators (total nitrogen, total alkaloids) ranged from 0.68 to 0.76, indicating a strong correlation between these two groups of metabolites. To further examine the regulatory direction, cross-pairing of the saccharide group with the nitrogen group was performed, and linear regression models incorporating interaction terms were separately constructed. The results showed that all four interaction term coefficients were negative and highly significant (p < 0.001), confirming that elevated saccharide concentrations significantly weaken the marginal contribution of nitrogenous substances to the sensory score, i.e., a clear antagonistic regulatory effect.

Finally, although the above eight key indicators are the primary driving factors for sensory irritation, the remaining 25 general indicators, though their individual feature importance values fall below the upper quartile threshold of 0.0010, collectively account for 52% of the total feature importance. Through complex synergistic effects and multi-component regulatory networks, these indicators still play a non-negligible role in shaping the sensory irritation of tobacco leaves.

### 3.6 Validation of key indicators

To further validate the core role and representativeness of the screened eight key chemical components (total nitrogen, total alkaloids, cryptochlorogenic acid, oleic acid + linolenic acid, reducing sugars, sugar-to-nitrogen ratio, Fru-Asp, and neochlorogenic acid) in determining tobacco leaf irritation, a binary RF model for high/low irritation was reconstructed using only these eight key chemical components. The trained model was applied to the independent test set for performance evaluation, and the confusion matrix results are shown in Table 6. The model achieved an accuracy of 79.69%, a precision of 81.58%, a recall of 83.78%, and an F1-score of 0.8267. Although these prediction indicators were slightly lower than those of the RF model constructed using the 33 features screened by the ReliefF algorithm (accuracy 84.38%), this reverse validation model still maintained a classification accuracy of nearly 80% using only a very small number of core features. This result fully demonstrates that these eight key chemical indicators effectively capture most of the core information determining the differences in sensory irritation of tobacco leaves, further confirming the accuracy of the feature importance analysis results.

**TABLE 6:** Classification confusion matrix of the RF model reconstructed based on eight key indicators on the test set.

True class	Predicted	
	Low irritation	High irritation
Low irritation	20	7
High irritation	6	31

## 4 Conclusion

1. Feature dimensionality reduction selection based on the ReliefF algorithm can effectively eliminate redundant features in chemical composition data, and using the selected 33 core features can significantly improve the classification performance and generalization ability of machine learning models.
2. The constructed ReliefF-RF binary classification model for tobacco leaf sensory irritation has excellent prediction and discrimination ability, achieving an accuracy of 84.38% on an independent test set, with comprehensive performance superior to the original RF model without feature screening and comparison models such as SVM and KNN.
3. The study identified eight key chemical components affecting high and low irritation of tobacco leaves. Increases in total alkaloids and total nitrogen exacerbate tobacco irritation; conversely, increases in reducing sugars, sugar-to-nitrogen ratio, cryptochlorogenic acid, neochlorogenic acid, oleic acid + linolenic acid, and Fru-Asp can effectively neutralize or reduce the irritation performance of tobacco leaves.

## 5 Limitation and future work

The ReliefF-RF classification model constructed in this study primarily serves as a predictive and screening tool linking chemical components to sensory irritation. Regarding its applicability domain, the current model is specifically optimized for the preliminary quality assessment and formulation screening of tobacco leaf raw materials. The eight key chemical determinants identified merely represent potential precursors to irritation, the actual sensory responses are primarily triggered by mainstream smoke aerosol components released during tobacco combustion and pyrolysis.

To bridge the current gap in mechanistic understanding, future research will focus on expanding this analytical framework by incorporating smoke chemical profiling. Integrating modern *in vitro* cellular assays, *in vivo* exposure models, and independent external validation using new factory batches will be essential to biologically validate these data-driven conclusions. Meanwhile, the specific mechanisms of these components targeting the oral cavity, pharynx, and lungs remain to be experimentally confirmed.

## Data Availability

The raw data supporting the conclusions of this article will be made available by the authors, without undue reservation.
